# Functional treatment for fractures to the base of the 5th metatarsal - influence of fracture location and fracture characteristics

**DOI:** 10.1186/s12891-017-1893-6

**Published:** 2017-12-16

**Authors:** Sebastian Felix Baumbach, Wolf Christian Prall, Michael Kramer, Mareen Braunstein, Wolfgang Böcker, Hans Polzer

**Affiliations:** Department of Trauma Surgery, Munich University Hospital, LMU, Nussbaumstr. 20, 80336 Munich, Germany

**Keywords:** Fifth metatarsal fracture, Lawrence and Botte, Functional treatment, Fracture characteristics, Outcome

## Abstract

**Background:**

Fractures to the base of the fifth metatarsal are common, but their treatment remains controversial. Especially for Lawrence and Botte (L&B) type II fractures, there is conflicting evidence and consequently no consensus. Further, many authors consider displacement, articular involvement, and number of fragments an indication for surgery, although evidence is missing. The aim of this study was to evaluate the outcome of functional treatment for all L&B type I and II fractures. Of special interest were the influence of (1) the fracture location (L&B type I vs. II) and (2) the fracture characteristics (displacement, intra-articular involvement, communition) on the subjective outcome.

**Methods:**

Retrospective registry study with a prospective follow-up. Patients with an acute, isolated, epi-metaphyseal fracture to the fifth metatarsal bone (L&B type I and II) treated by full weightbearing with a minimum follow-up of 6 months were included. Fracture location (L&B type I and II) and characteristics (displacement <2 mm or >2 mm, intra-articular involvement, and number of fragments) were assessed. Outcome parameters were return to work, return to sports, VAS-FA, and SF-12. The influence of the fracture (1) location and (2) -characteristics on these parameters was tested.

**Results:**

Thirty-nine patients (40 ± 15 years, 56% female) were enrolled with a mean follow-up of 22 ± 10 months. L&B type I fractures occurred in 59%, type II in 41%. Thirty-one percent of all fractures were dislocated, 74% intra-articular, and 41% multi-fragmentary. Patients returned to work after 17 ± 12 days, to sports after 53 ± 22 days. The VAS-FA score at the final follow-up was 96 ± 4, SF-12 PCS score 57 ± 5 and MCS score 51 ± 8. No complications were reported, no patient required surgery. None of the assessed outcome parameters differed significantly between (1) the different fracture locations (L&B type I vs. II) or (2) the different fracture characteristics (displacement, intra-articular involvement, and number of fragments).

**Conclusions:**

(1) Both, L&B I and II fractures featured excellent results with immediate full weightbearing. Consequently, L&B type I and II fractures should be summarized as epi-metaphyseal fractures. (2) Fracture displacement, articular involvement, and number of fragments did not influence the outcome. Therefore, functional treatment should be recommended for all epi-metaphyseal fractures.

## Background

**Fig. 1:**
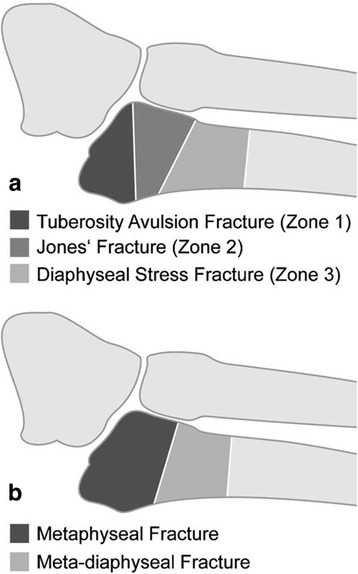
Illustration of the Lawrence and Botte [3] as well as the Polzer [5] classifications. **a** Lawrence and Botte classification; **b** Polzer classification; Reprinted from Injury, Vol 43, Polzer H, Polzer S, Mutschler W, Prall WC, Acute fractures to the proximal fifth metatarsal bone: development of classification and treatment recommendations based on the current evidence, pp. 1626–1632, Copyright (2012), with permission from Elsevier

Treatment recommendations for fractures to the base of the fifth metatarsal (MTV) are still a matter of debate. Possible causes are missing evidence, various fracture classifications, and diverging definitions for the term “Jones fracture” [1–4]. The most widely accepted classification was published by Lawrence and Botte [3] (L&B) in 1993. They differentiated tuberosity avulsion fractures (type I), Jones’ fractures (type II) and diaphyseal stress fractures (type III) (Fig. 1). A recent systematic literature review [5] evaluated the validity of the classification system and treatment recommendations by Lawrence and Botte. Overall the level of evidence available was moderate. Based on this evidence a treatment-oriented adaptation of the L&B classification was concluded. In summary, L&B type I and II fractures should not be differentiated but be summarized as epi-metaphyseal fractures, as both apparently heal well when treated functionally. Although these recommendations are based on strong evidence for L&B type I fractures, only little evidence is available for L&B type II fractures. In contrast, strong evidence is available in favour of surgical treatment for L&B type III fractures (meta-diaphyseal fractures). Furthermore, it remains largely unknown, whether fracture characteristics, i.e. displacement, articular involvement, number of fragments, negatively influence the outcome of functional treatment and therefore require surgery.

Therefore, the aim of this study was to evaluate the outcome of functional treatment for all L&B type I and II fractures. The following two hypotheses were tested:

1. The fracture location (L&B type I vs. II) does influence the outcome.
2. The fracture characteristics (displacement, intra-articular involvement, communition) affect the outcome.

## Methods

### Study design

Retrospective registry study with a prospective follow up. The study was approved by the local ethic committee (# 541–14).

### Patient selection

The study was conducted by the Division of Foot and Ankle Surgery at the University Hospital of Munich (LMU). Patients suffering an isolated epi-metaphyseal fracture to the proximal fifth metatarsal bone (L&B type I or II, Fig. 1) between Jan. 1st 2012 and Oct. 1st 2014 were identified retrospectively using the clinical and radiographic database of the Munich University Hospital. The clinical database was searched for patients diagnosed with a metatarsal fracture (ICD-10 Vs. 2013: S92.3). The radiographic database was searched for the terms: Metatarsal AND fracture AND (5 OR V) OR Jones. All fractures were reviewed independently by two investigators (SFB, HP). First, isolated fractures to the fifth metatarsal were identified. Then, fractures located distal to the fourth–fifth intermetatarsal articulation were excluded. All remaining fractures were located at the base of the fifth metatarsal (L&B type I and II) not extending beyond the distal end of the fourth–fifth intermetatarsal articulation (L&B type III). These patients were invited for a final follow-up visit. Only patients meeting all inclusion criteria, including the final follow-up visit, were enrolled. Eligibility criteria are stated in detail in Table 1.

**Table 1:** Inclusion and exclusion criteria

Inclusion criteria	Exclusion criteria
Age ≥ 18 years	Age < 18 years
Isolated, traumatic, epi-metaphyseal fractures to the proximal fifth metatarsal (L&B type I & II)	Meta-diaphyseal- (L&B type III), shaft-, and distal fractures to the fifth metatarsal
Follow-up of at least 6 month	Any further injuries
	Non-traumatic or any pathological fractures
	Time to treatment >6 weeks
	Missing initial radiographs

**Fig. 2:**
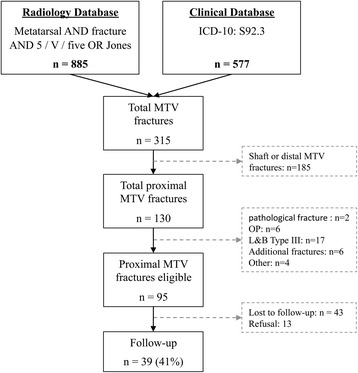
Flow chart depicting the patient selection. MTV: Fifth metatarsal; n = Number; L&B: Lawrence and Botte; OP: Operation

The results of the search strategy are outlined in Fig. 2. The database search identified 315 fractures affecting the fifth metatarsal bone. One hundred thirty were located at the proximal end of the fifth metatarsal. In total 95 fractures met the inclusion criteria. Thirty-nine patients (41%) completed the final follow-up and were therefore included in the final analysis. Their mean age was 40 ± 15 years, 56% were female.

### Treatment regimen

Patients with acute fractures to the proximal fifth metatarsal first presented to the emergency department and were then transferred to the outpatient clinic of the Division of Foot and Ankle Surgery. After informed consent, functional treatment was initiated. The treatment recommendations have been described in detail earlier [5]. In brief, all patients were instructed to wear shoes with a stiff sole and conduct weightbearing as tolerated. In case pain limited immediate full weightbearing, partial weightbearing on crutches was conducted. Full weightbearing was recommended as soon as possible, the latest after 2 weeks. In case of severe swelling the foot was immobilized using a walker or a short leg cast for a maximum of 2 weeks. Return to work and sports was encouraged as soon as possible. Radiographic follow-up was performed in case of prolonged symptoms of more than 6 weeks only. If symptoms subsided within 6 weeks, no radiographic follow-up was performed. This treatment regimen was conducted for all epi-metaphyseal fractures (L&B type I & II), not extending beyond the distal end of fourth–fifth intermetatarsal articulation (L&B type III), regardless of the number of fragments, displacement and intraarticular involvement.

### Data collection

Data were collected retrospectively based on patient records and prospectively based on a follow-up visit or phone interview. Table 2 lists the variables collected. Fracture classification and characteristics were assessed on the initial radiographs by two independent investigators (SFB, HP). Disagreement was resolved by discussion. Fractures were first classified per Lawrence and Botte type I or II [3]. Then, the following fracture characteristics were assessed: Displacement (none, ≤2 mm, >2 mm), intra-articular involvement (binary), and number of fragments (two- or multiple). Fractures were classified intra-articular if the articulation between the metatarsal and cuboid bone was affected. Finally, if available, radiographic follow-ups after 6 weeks were evaluated. Patient records were screened for complications as well as return to work and sports. In order to evaluate the mid-term results, patient rated outcome measures were assessed at the final follow-up visit at least 6 months after the trauma.

**Table 2:** Variables assessed based on the patient records and follow-up

Patient records	Age, Sex
	Fracture location: Lawrence and Botte type I and II [3] Fracture characteristics: Number of fragments, displacement, articular involvement
	Complications Return to work Return to sports Radiographic follow-up (when available)
Follow-up	VAS-FA [6] SF-12v2

### Outcome variables

The primary outcome parameter was return to work. Secondary outcome parameters were return to sports, the Visual Analog Scale Foot and Ankle (VAS-FA) [6] and the 12-item short-form (SF-12v2). The VAS-FA was evaluated using the developers’ analysis instrument [6]. The VAS-FA is a validated, subjective, VAS based foot and ankle outcome score. It comprises of 20 items, grouped into three categories (function, pain, other complaints) [7, 8]. Score values for the overall and subgroup scores range between 0 and 100 points, with higher scores indicating a better outcome. The SF-12 score was analysed using the manufactures analytic tool-box (license number QM027870). The SF-12v2 is a short version of the SF-36 Health Survey, measuring eight domains. These are consolidated into two meta-scores: The Mental Component Summary (MCS) and the Physical Component Summary (PCS). The MCS and PCS scores range between 0 to 100, with a score of 50 being representative for a sample population.

### Data analysis and statistics

Data analysis was conducted to assess the influence of (1) the fracture location (L&B type I vs. II) and (2) the fracture characteristics (displacement, intra-articular involvement, and number of fragments) on the outcome variables outlined above. Statistics applied were standard descriptive statistics, independent T-tests, and Chi-Square-test, were appropriate. Furthermore, the influence of the fracture classification and characteristics (independent variables) on the outcome parameters (dependent variable) were assessed using a linear regression model (Enter Method). Results are indicated as means ± standard deviation (SD). Due to multiple testing, an alpha-level correction (Bonferroni) was conducted (*p* < 0.007). Statistics were computed using SPSS Vs. 21 (IBM Company).

## Results

### Fracture classification and characteristics

The left proximal MTV was fractured in 49%. Lawrence and Botte type I fractures occurred in 59% of the patients. Out of all fractures, 31% were dislocated (>2 mm), 74% intra-articular, and 41% multi-fragmentary (Table 3). Intra-articular fractures (fifth metatarso-cuboid joint) occurred significantly more often in L&B type I compared to type II fractures (*p* = 0.004). No significant differences were observed for displacement or number of fragments between both fracture types. No significant age, side, or gender differences were found for any of these parameters.

**Table 3:** Fracture characteristics per L&B Zone I and II fractures

	Number	Dislocated	Intra-articular	Multi-fragmentary
Total	39	31%	74%	41%
L&B type I	23 (59%)	26%	91%	39%
L&B type II	16 (41%)	38%	50%	44%
*p*-value		0.447	0.004	0.773

*L&B* Lawrence and Botte

### Treatment details

All patients were treated according to the regimen outlined above. Twenty-eight percent of patients bore weight fully in a hard-soled shoe immediately. The remaining patients were temporarily immobilized for a maximum of 2 weeks (cast: 8%, walking boot: 54%, short walking boot: 10%). The mean number of follow-up visits at our outpatient clinic was 1.3 ± 1.6 (range: 0–6). This figure does not include the final follow up visit, which was conducted to assess the mid-term results.

### Outcome

The mean follow-up was 22 ± 10 month (6–40 month). The patients returned to work after 17 ± 12 days (range: 0–56 days), and to previous sports levels after 53 ± 22 days (range: 21–100) on average. At final follow-up, the overall VAS-FA score was 96 ± 4, the subscale pain 95 ± 7, and the subscale function 97 ± 4. The mean SF-12 PCS score was 57 ± 5 and the MCS score 51 ± 8. No complications were reported, no patient required surgery. Seven patients (18%) suffered symptoms after 6 weeks. Only these patients received a radiographic follow-up. All these follow-up radiographs were unsuspicious, showing bony union in all cases. Patients receiving follow-up radiographs did not differ in any respect (fracture location, −characteristics, or outcome variables) to those with no follow-up radiographs. No complications were identified.

### Influence of fracture location

The above outlined outcome variables (return to work, return to sports, VAS-FA, SF-12) were analysed separately for each fracture location (L&B type I and II). An independent students t-test revealed no significant difference for these outcome variables between the two fracture locations (Table 4). The linear regression model also proofed no significant influence of the fracture location on any of the outcome parameters.

**Table 4:** Fracture type and outcome variables at final follow-up

	Return to work [d]	Return to sports [d]	VAS Overall	VAS Pain	VAS Function	SF-12 PCS	SF-12 MCS
Total	17 ± 12	53 ± 22	96 ± 4	95 ± 7	97 ± 4	57 ± 5	51 ± 8
L&B type I	15 ± 10	47 ± 19	97 ± 3	96 ± 6	98 ± 3	58 ± 3	51 ± 7
L&B type II	20 ± 15	63 ± 25	95 ± 4	92 ± 7	96 ± 5	55 ± 7	51 ± 9
p-value	0.194	0.040	0.098	0.051	0.069	0.133	0.915

*d* days, *L&B* Lawrence and Botte [3], *VAS* VAS-FA [6], *SF-12 PCS* SF-12 Physical Health Composite Scores, *SF-12 MCS* Mental Health Composite Scores

### Influence of fracture characteristics

A similar analysis was conducted to assess the influence of the fracture characteristics (displacement, intra-articular involvement, and number of fragments) on all outcome variables (Table 5). None of the fracture characteristics individually had a significant influence on any of the outcome parameters. Similar, the linear regression model showed no significant effect of the fracture characteristics on the outcome scores.

**Table 5:** Fracture characteristics and outcome parameters at final follow-up

	Return to work [d]	Return to sports [d]	VAS Overall	VAS Pain	VAS Function	SF-12 PCS	SF-12 MCS
Total	17 ± 12	53 ± 22	96 ± 4	95 ± 7	97 ± 4	57 ± 5	51 ± 8
Non-dislocated	18 ± 12	51 ± 23	96 ± 4	95 ± 7	97 ± 4	56 ± 6	51 ± 7
Dislocated	15 ± 12	57 ± 22	97 ± 3	95 ± 7	98 ± 3	58 ± 3	51 ± 9
p-value	0.502	0.536	0.323	0.987	0.439	0.220	0.876
Extra-articular	17 ± 13	60 ± 28	95 ± 5	92 ± 9	96 ± 5	56 ± 6	50 ± 11
Intra-articular	17 ± 12	51 ± 20	97 ± 3	96 ± 6	98 ± 4	57 ± 5	52 ± 6
p-value	0.970	0.343	0.340	0.202	0.275	0.546	0.604
Two fragments	16 ± 10	52 ± 25	96 ± 4	95 ± 7	97 ± 4	56 ± 4	53 ± 8
Multi fragmentary	18 ± 15	54 ± 20	96 ± 4	95 ± 7	97 ± 4	57 ± 7	49 ± 8
*p*-value	0.682	0.833	0.991	0.971	0.835	0.760	0.214

*d* days, *VAS* VAS-FA [6], *SF-12 PCS* SF-12 Physical Health Composite Scores, *SF-12 MCS* SF-12 Mental Health Composite Scores

## Discussion

Whereas there is a broad consensus on functional treatment for non-displaced L&B type I fractures, limited evidence is available for the best treatment for L&B type II fractures [9–17]. Today, few studies report promising results following functional treatment while other authors argue for operative treatment [18–21]. A major reason for these conflicting recommendations is the inconsistent use of the term “Jones fracture” for both L&B type II and III fractures [22–24]. An example for the confusion resulting from this lack of definition is the systematic review by Roche et al. [25], analysing the outcome of “Jones fractures” in 26 studies. When looking at these studies in detail, the great majority analysed L&B type III fractures. Some did not clearly define the fracture types and only one study clearly included L&B type II fractures. In consequence, the actual treatment recommendation for type II fractures remains unclear.

In the herein presented study, early functional treatment of all L&B type I and II fractures lead to excellent results (Tbl. 4). Both, return to work and sports were comparable to previous studies including L&B type I fractures only [12, 14, 16, 26]. The mean foot specific outcome score (VAS-FA) for all fractures was similar to healthy individuals (94.5 ± 8.2 Points) [27]. The participants’ quality of life scores (SF-12: PCS and MCS) were slightly higher than the populations’ average. Finally, our treatment regimen did not result in any complications or conversion of treatment. When comparing L&B type I and II fractures no significant differences could be detected for any outcome parameter within a follow-up of 22 ± 10 months.

The few studies available for type II fractures demonstrated comparable results to the herein presented findings. Still they are inherent of shortcomings that need to be discussed. Bigsby et al. [19] reported on the outcome of 62 type I and 26 type II fractures. No differences were found for the Foot Function Index (FFI) and the Short Form 36 between type I and II fractures. Unfortunately, no standardized treatment regimen was applied. Konkel et al. [28] treated 35 type I and 10 type II fractures nonoperatively. Treatment varied from no treatment to immobilization in a short leg cast. In average patients required 3.5 months to resume full duty. The orthesis, cast or shoe was applied for a minimum of 6 weeks. This long immobilization might have contributed to the prolonged time of recovery. Nevertheless, 100% of the patients were satisfied with the result. Van Aaken et al. [18] applied functional treatment for 15 type I and 8 type II fractures with an elastic dressing. The mean time to return to work was 21 days for patients with type I fractures compared to surprisingly 4 days for patients with type II fractures. Taken together, type II fractures can be treated functionally with an excellent clinical outcome, comparable to L&B type I fractures.

Further, many authors postulate that displaced (>2 mm), multifragmentary, or intra-articular fractures necessitate operative treatment. Almost all of these recommendations are solely based on the authors’ opinion, but not on evidence [3, 10, 29–33]. Therefore, the second aim of this study was to evaluate the influence of these aspects on the outcome of functionally treated L&B type I and II fractures. None of the fracture characteristics analysed, namely fracture displacement greater 2 mm, articular involvement, or comminution, affected any of the outcome parameters assessed.

To the authors’ knowledge, only two studies report data regarding the impact of intra-articular involvement and displacement on the clinical outcome [16, 17]. Egol et al. [16] treated L&B type I fractures by immediate weightbearing as tolerated. Out of these, 50% were intra-articular and 32% displaced (>2 mm). The average time to return to work was 22 days. Comparing intra- to extra-articular fractures and non-displaced to displaced fractures, no significant differences could be observed for any of the outcome parameters (SMFA pain, VAS). Tahririan et al. [17] treated 143 patients with a fracture to the base of the fifth metatarsal (L&B type I, II, III) with a short leg cast for 6 weeks. The average AOFAS score after 20 weeks for all fractures was 93 with a 95% confidence interval of 92–94. The multivariate analysis revealed that displacement, patient weight, type III fractures, diabetes and female gender were associated with a poorer AOFAS. One should keep in mind, that the AOFAS score, the only outcome parameter assessed, has been proven to be poorly valid and the minimal important clinical difference of this score is unknown [34]. Moreover, the average AOFAS in this study was extremely high with a remarkably narrow CI suggesting an excellent outcome for all fractures. Finally, the results of the statistical analysis are not comprehensible, as the authors did not present any data in detail. All in all, the data presented in our study argue for functional treatment of all L&B type I and II fractures, independent of displacement, articular involvement, or comminution.

Several limitations and strengths have to be discussed. First, the most pronounced limitation is the initial retrospective data assessment. Second, the final follow-up rate is limited to 41%. Consequently, it is unclear what happened to the patients lost to follow up, whether they received surgery elsewhere, suffered inferior clinical results or were in line with the patients included in this study. Still, this follow-up rate is compare to previous studies [35–37]. Third, patient specific factors such as intrinsic bone disorders or metabolic disorders were not assessed. Fourth, despite a mean follow-up of almost 2 years, refractures have been reported to occur even after that time range [38]. Still, most recent studies report low re-fracture rates in operatively treated patients initially suffering a stress fracture (L&B type III) [39] or athletes [21, 38, 40]. The risk for re-fracture after conservative treatment for L&B type I and II fractures remains unknown. A final limitation could be, a missing radiographic follow up for all patients. In accordance with literature, radiographic follow up was only conducted in case of prolonged symptoms of more than 6 weeks. In these patients, no non-union was observed. Still, asymptomatic non-unions could have been missed. As an asymptomatic non-union does not require any further treatment the authors would not consider this a complication. Therefore, we are convinced, that the missing radiographic follow-up of asymptomatic patients should not be considered a limitation. Contrariwise, this regime drastically reduces the number of follow-up visits.

Strengths of this study were the use of two validated patient rated outcome measures, one specific for foot and ankle disorders, the other a commonly used quality of life score [6, 27]. Furthermore, the fracture characteristics were assessed by two independent investigators and a mean, prospective follow up of almost 2 years was reached. Finally, a detailed workup of various factors possibly influencing the results was presented.

## Conclusions

In conclusion, functional treatment leads to excellent clinical results for both, L&B type I and type II fractures. (1) As both fracture locations did not differ for outcome, they should not be delineated, but rather be summarized as epi-metaphyseal. (2) Fracture displacement greater 2 mm, intra-articular involvement, and comminution did not affect the outcome. Therefore, functional treatment should be applied to all epi-metaphyseal fractures, even when displaced, intra-articular or comminuted.

## Abbreviations

%: Percent
<: Smaller than
>: Greater than
±: Plus / minus
≤: Smaller or equal
d: Days
Fig.: Fig.
ICD-10: International Statistical Classification of Diseases and Related Health Problems by the World Health Organization
L&B: Lawrence and Botte
MCS: Mental component summary
mm: Millimetre
MTV: Fifth metatarsal bone
p: *p*-value
PCS: Physical component summary
SD: Standard deviation
SF-12: 12-item short-form of the SF-36 Health Survey
VAS-FA: Visual analog scale foot and ankle
Vs.: Versus
